# Investment by maternal grandmother buffers children against the impacts of adverse early life experiences

**DOI:** 10.1038/s41598-024-56760-5

**Published:** 2024-03-21

**Authors:** Samuli Helle, Antti O. Tanskanen, David A. Coall, Gretchen Perry, Martin Daly, Mirkka Danielsbacka

**Affiliations:** 1 INVEST Research Flagship Centre, University of Turku, Turku, Finland; 2 Population Research Institute, Väestöliitto, Helsinki, Finland; 3 School of Medical and Health Sciences, Edith Cowan University, Joondalup, Australia; 4 School of Social Work, University of Canterbury, Christchurch, New Zealand; 5 Department of Psychology, Neuroscience and Behaviour, McMaster University, Hamilton, ON Canada; 6 Department of Social Research, University of Turku, Turku, Finland

**Keywords:** Evolution, Psychology

## Abstract

Exogenous shocks during sensitive periods of development can have long-lasting effects on adult phenotypes including behavior, survival and reproduction. Cooperative breeding, such as grandparental care in humans and some other mammal species, is believed to have evolved partly in order to cope with challenging environments. Nevertheless, studies addressing whether grandparental investment can buffer the development of grandchildren from multiple adversities early in life are few and have provided mixed results, perhaps owing to difficulties drawing causal inferences from non-experimental data. Using population-based data of English and Welsh adolescents (sample size ranging from 817 to 1197), we examined whether grandparental investment reduces emotional and behavioral problems in children resulting from facing multiple adverse early life experiences (AELEs), by employing instrumental variable regression in a Bayesian structural equation modeling framework to better justify causal interpretations of the results. When children had faced multiple AELEs, the investment of maternal grandmothers reduced, but could not fully erase, their emotional and behavioral problems. No such result was observed in the case of the investment of other grandparent types. These findings indicate that in adverse environmental conditions the investment of maternal grandmothers can improve child wellbeing.

## Introduction

A large body of literature has demonstrated how stressful physical and social early life environments, starting in utero, can shape later life fitness-related traits in both human and non-human species^1–3^. Some suggest that in long-lived species like humans, such adverse early life conditions can inflict constraints and trade-offs that impair an individual’s capacity to perform optimally in later life^4^ while others propose that at least some of these effects are adaptive rather than maladaptive^5,6^. A recent theoretical perspective emphasizes the impacts of specifically social aspects of the environment (e.g., helping behaviour and parental care) experienced during development on outcomes such as an individual’s later prosocial behavior^7^. Furthermore, because cooperative breeding and non-kin cooperation probably have evolutionary roots in coping with unpredictable and stressful environments^8,9^, and humans closely resemble cooperatively breeding species^10^, it is possible that investment by grandparents in their grandchildren (i.e., any costly support or resources allocated towards grandchildren either directly or indirectly such as via a grandchild’s parents^11^) can play important roles in unfavorable conditions, such as when children suffer from adverse family conditions^12^.

On average, grandparents transmit 25% of their genes to their grandchildren and thus can gain fitness benefits by investing in them^13^, but grandparental investment is relatively rare in non-human species. Species where grandmothers occasionally care for their grandoffspring include some primates, elephants, birds, and whales^14–21^, but care by grandfathers may be non-existent in non-human species^22^.

In traditional and historical populations (or subsistence societies) with multiple environmental stressors and high child mortality rates, the presence of grandmothers has often been found to be associated with improved early-life survival of children^23–31^. In most cases the presence of grandfathers has not been found to be similarly associated with improved grandchild survival in past populations^32^ and in some cases the association is actually negative^33^. These findings are in line with the grandmother hypothesis arguing that the long post-reproductive lifespan of human females has evolved because it particularly benefits the fitness of older women (compared to men) to invest resources to support reproductive efforts of their children and survival of grandchildren^34–36^. Although the grandmother hypothesis has been challenged^37,38^, it is clear that in subsistence societies, grandmothers have often been able to increase the survivorship of their grandchildren and the reproductive output of their daughters and daughters-in-law^23,26^.

Just as investment by kin is ubiquitous, it is also unequal^39^. There is evidence that in traditional and historical populations, maternal relatives have been more important for grandchild survival than paternal ones^12,23,27^. Due to pregnancy and lactation, having a child is energetically more costly for women than for men, meaning that women have a greater obligatory investment in the development of a child than men^40^. This asymmetry between women and men may also lead to an asymmetry in the inclinations of maternal and paternal grandparents because a contribution to the health and wellbeing of one’s daughter has greater expected fitness benefits than a like contribution to one's daughter-in-law^41^. Moreover, because of paternity uncertainty, maternal grandmothers (MGMs) are the only ones who can be certain of their genetical relatedness to their grandchildren, while maternal grandfathers (MGFs) and paternal grandmothers (PGMs) both have one, and paternal grandfathers (PGFs) have two uncertain links of paternity^42,43^. Hence, even though the kinship coefficient (or the average degree of genetic relatedness) is similar among the four grandparent types, due to paternity uncertainty there could be systematic differences in the costs and benefits of grandparental investment between the grandparent types^44–46^.

Although grandparents may have been important alloparents in past populations, grandparental investment should not be considered a “relic of the past” because there are several reasons why grandparents continue to be an important part of families in contemporary affluent societies^47^. First, due to increased life expectancy, the proportion of children with living and healthy grandparents is increasing^11^. Second, as the average lifespan has increased, grandparents and grandchildren have more years of shared lifetime together than ever before^48^. Third, grandparents today have fewer grandchildren than before and thus may be able to channel more investment into each grandchild^49^. Fourth, as the number of working mothers in contemporary affluent societies has substantially increased, there is also increased need for grandparental investment^50^. Fifth, because of increased divorce rates and non-marital births, grandparental investment is often a necessity for many single-parents struggling to manage their everyday lives^51^. Hence, while in contemporary settings in the rich world grandparents are no longer needed to keep their grandchildren alive, grandparental investment may still improve child wellbeing, for example, by decreasing emotional and behavioral problems of children^11^.

Prior studies considering the association between grandparental investment and child wellbeing in contemporary western societies have, however, provided mixed results. Some have found that maternal grandparental investment is associated with decreased emotional and behavioral problems in children^52,53^, but others have argued that grandparental investment may not be causally linked with child wellbeing^54^. Also, studies using other measures for child outcomes, such as early-year development or educational achievements have provided mixed findings^12,55^.

These inconsistencies could stem from the shortcomings of how environmental stressors have been considered in prior studies. Some studies from present-day societies have found that during times of parental separation, mother’s depression, unemployment or economic hardship, grandparent investment is associated with improved child wellbeing^52,56–61^. In addition to that these studies have not applied causal methodologies, a key limitation of these studies is that they have considered only one or a few adversities at a time. When the aim is to study adaptive kin effects it is important to take multiple adversities into account to effectively measure total environmental stress^62^. According to prior findings, particularly MGMs may increase their investment in grandchildren in the case of increased need for help^63,64^.

Only three studies, all relying on correlational evidence, so far have taken cumulative adverse early life events (AELEs) into account when assessing whether grandparental investment is associated with decreased emotional and behavioral problems in adolescents. The first such study found that the investment of the closest grandparent buffered the influence of adversities on grandchildren’s psychopathology in England and Wales^65^, but such an association was not found in a following study in Malaysia^66^. The third study reported a protective role of the investment of the closest grandparent in South Africa, but only in girls^67^. In these studies, only the grandparent with the highest investment frequency was incorporated into the analyses, which may create biases^12^. This means that we do not currently know whether the buffering role of grandparents in contemporary wealthy societies varies by grandparent type as suggested by evolutionary reasoning and prior studies.

It is well known that children who suffer from cumulative AELEs also have more emotional and behavioral problems^68^. It is also evident that supportive caregiving can protect children from early adversity-induced psychopathology^69^. Based on the grandmother hypothesis, sex-specific reproductive strategies and paternity uncertainty, we hypothesize that maternal grandmothers should have the strongest influence of all grandparents in reducing the negative consequences of growing up in adverse environments. This expectation is corroborated by a recent study showing that MGMs did not reduce their investment when their grandchild experienced several AELEs, whereas other grandparent types did^70^.

We use population-based data from English and Welsh adolescents aged 11–16 years. As a measure of children’s emotional and behavioral problems, we used a total score of the Strengths and Difficulties Questionnaire (SDQ), which is commonly used to measure difficulties in children aged 4–17 years^71^ and has previously been shown to be positively correlated with the number of stressful events in these data^65^. Since it is unrealistic to assume that there is no confounding among the variables studied here, or that we could adjust for all potential confounders by using the current data, analyses based on traditional regression modeling most likely fall short on permitting justified causal inferences owing to endogeneity, that is, to the fact that the model error term is likely to be correlated with the independent variable due to omitted causes, measurement error in the independent variable or simultaneity and even reversed causality^72^. We therefore take advantage of instrumental variable regression, fitted in a Bayesian structural equation modeling (BSEM) framework, to obtain more causally interpretable estimates. While commonly used in econometrics^73^ and social sciences^74^, instrumental variable regression has only very recently become part of the statistical toolbox of psychologists^75^ and biologists^76,77^. The need for causal approaches has recently been called for in the grandparental literature^78^.

## Methods

### Data

The current research used the Involved Grandparenting and Child Well-Being 2007 survey, conducted by GfK National Opinion Polls, which is a nationally representative sample of English and Welsh adolescents aged 11–16^79–81^. Children of randomly-chosen classrooms in selected schools completed the questionnaire (response rate was 68%) and the original sample included 1566 adolescents^79,82^. Children were asked questions for only those grandparents who were still alive. Hence, only those respondents who had at least one living grandparent (*n* = 1488) were considered in the analyses. As commonly done in previous research, we also excluded from the analyses those children who were co-residing with their grandparents (*n* = 58). This was done because we cannot separate the cases where grandparents were the sole caretakers of grandchildren (in which case their investment is much more obligatory) from those cases of three-generation households. The total number of children included in the analyses ranged from 817 to 1197, depending on the grandparent type considered.

*Grandchild’s emotional and behavioral problems* We used The Strengths and Difficulties Questionnaire (SDQ), which is a standard behavioral screening for children aged 4–17 years^71,83^. SDQ may help in predicting behavioral problems like attention deficit hyperactivity disorder (ADHD) in children^84^ and prevalence of psychopathological disorders in community samples^83^. It consists of 5 subscales, namely emotional symptoms, conduct problems, hyperactivity/inattention, peer relationship problems and prosocial behavior, each having 5 items. Of these 5 subscales, 4 subscales excluding prosocial behavior, are used to calculate a total SDQ score (the mean score ranging from 12.59 to 12.91 and its SD from 5.55 to 5.71), which we use as the outcome variable in the current study (Table 1). A higher total score indicates more emotional and behavioral problems, the range being 0–33^71^.

**Table 1:** Selected results of instrumental variable regression models on the effect of grandparental investment (GI) on grandchild’s total The Strengths and Difficulties Questionnaire (SDQ) score, moderated by adverse early life experiences (AELEs) for different grandparental types.

	median	95% CI	One-tailed probability
MGMs			
Granparent's investment (GI)	2.476	0.923, 4.012	0.001
AELEs	3.196	1.493, 4.873	0.000
GI × AELEs	− 0.839	− 1.484, − 0.212	0.005
MGFs			
Granparent's investment (GI)	1.037	− 0.761, 2.905	0.128
AELEs	1.547	− 0.415, 3.518	0.062
GI × AELEs	− 0.227	− 1.008, 0.577	0.284
PGMs			
Granparent's investment (GI)	1.699	− 0.700, 4.229	0.081
AELEs	2.209	− 0.164, 4.739	0.034
GI × AELEs	− 0.599	− 1.711, 0.447	0.128
PGFs			
Granparent's investment (GI)	1.730	− 1.349, 4.853	0.125
AELEs	1.921	− 1.263, 5.129	0.107
GI × AELEs	− 0.450	− 1.987, 1.019	0.266

95% CI denotes a 95% credibility interval of the posterior median of coefficients after multiple imputation to handle missing data. For a positive posterior median, one-tailed probability gives the proportion of posterior distribution that is below zero, and for a negative posterior median the proportion of posterior distribution that is above zero is given.

*Measurement of a grandchild’s adverse early life experiences (AELEs)* We applied the distal adverse life events scale of Tiet et al.^85^ to record the number of adverse life events experienced prior to the previous year in order to measure the grandchildren’s AELEs. The Adverse Life Event scale consists of 25 possible adverse events that children have little or no control over. However, the original scale included several events that may not have been severe enough to have a strong enough influence on grandparental investment^70^. Hence, we included only the following events (answered yes/no) with specific reference to the grandchild or her/his family to calculate the number of AELEs for each grandchild in earlier life: “someone in the family died”, “there was a negative change in parent's financial situation”, “family had drug/alcohol problem”, “respondent got seriously sick or injured”, “respondent was a victim of crime/violence/assault”, “parents separated or divorced”, and “one of the parents went to jail”. Moreover, we also included the question “have you ever qualified for free school meals (even if not taken)?”. As children from low-income families receive free school meals in the UK, this variable indicates the financial conditions of the family. Thus, the resultant composite index for AELEs was the sum of eight events over which adolescents have little or no control, and higher scores mean more adverse early life experiences (the mean score ranging from 1.50 to 1.55 across different grandparent types, its SD being 1.39). Please note that although these data did not provide any direct measures of parental investment in children, most of the questions used to measure AELEs do indicate reduced parental ability to provide for their children.

*Grandparental investment.* In order to measure grandparental investment in grandchildren, we used questions developed by Elder and Conger^86^, included in The Involved Grandparenting and Child Well-Being 2007 survey. From the list of all questions available, we chose four that directly measured grandparental investment. These were “how often do you see them” (Q15), “their grandparents had looked after them” (Q26), “they could depend on their grandparents” (Q27), and “provided financial assistance or help” (Q38). Question Q26 was reverse-scaled to match the meaning and ordering of other scales. Questions Q26, Q27 and Q38 were measured on a 4-point Likert-type scale ranging from 1 = *not at all/never* to 4 = *a lot/every day* and question Q15 was measured on a 3-point Likert-type scale ranging from 1 = *never* to 3 = *usually.* For descriptive statistics, please see supplementary materials Table S4.

### Ethical approval and consent to participate

The collection of these data was approved, and the research was performed in accordance with the guidelines of the University of Oxford Research Committee. All the participants and their parents gave written consent to participate in the study in accordance with the Declaration of Helsinki.

### Statistical analysis

In order to obtain more causally defendable results, we applied instrumental variable regression in a structural equation modeling (SEM) framework. Instruments are secondary variables in the analysis (i.e., not themselves of scientific interest) that act as predictors of independent variables in the model, and it is the causal effects of these independent variables on outcomes that are of scientific interest^73^. In its most common use, instrumental variable regression relies on a multi-stage mean and covariance structure approach, estimated non-iteratively using two-stage least squares (2SLS). However, the analysis can also be readily performed using a structural equation modeling (SEM) framework with maximum likelihood or Bayesian estimation where it is possible to model error covariances between the independent and outcome variables that represent e.g. all the potential omitted confounding variables^75,87^. Causal identification in instrumental variable estimation is based on three main assumptions: i) instruments must be strongly associated (no causality is needed though) with the independent variable (i.e., relevance criterion), (ii) instruments must not have direct effects on the outcome (i.e., exclusion criterion), and (iii) instruments and the outcome do not share common causes. The assumptions (ii) and (iii) cannot be empirically verified since the error of the outcome is by definition unobserved. Some evidence supporting these assumptions can however be obtained by evaluating the association between the instruments and the outcome when multiple instruments are available. Note that it is not possible to simultaneously estimate both the direct effect of instruments on the outcome and their covariance arising from shared unmeasured causes affecting both the instrument and the outcome because such a model is never identified. However, although the underlying causal reasoning between these two differs, modelling either a direct effect (i.e., a regression) or a covariance will result in equivalent statistical models (i.e., they are indistinguishable from data) and will thus provide similar support for both the assumptions (ii) and (iii). Furthermore, as in regular regression, all associations are assumed to be linear and homoscedastic^75^. At least one instrument per independent variable is needed for the instrumental variable regression model to be identified but more instruments per independent variable (i.e., over-identification) are required to test the exclusion criterion [here basically including both assumptions ii) and iii)]. The causal effect identified in instrumental variable regression is the local average causal effect (i.e., the effect of an independent variable on the outcome among the cases affected by the instruments used)^88^. The sample sizes available here should enable good performance of instrumental variable estimators^87^.

One of the main difficulties with instrumental variable regression is finding appropriate instrumental variables that satisfy the assumptions described above. This is also the case with the current data that had not been collected with the instrumental variable regression in mind. Out of the very few potential variables that might serve as instrumental variables for grandparental investment in grandchildren, we chose living distance between the grandchild and the given grandparent because living distance is very unlikely to have a direct effect on grandchildren’s emotional and behavioral problems and because the grandparent’s living distance and investment showed reasonably high correlations (MGMs: *r* = 0.71: MGFs: *r* = 0.70; PGMs: *r* = 0.55; PGFs: *r* = 0.46). Furthermore, this relevance criterion should be tested by evaluating whether the instrument is weak or strong in predicting grandparental investment, beyond mere statistical significance^75^. Commonly, an *F* statistic > 10 in the case of a single independent variable instrumented is considered strong^89^. The higher the *F*-statistic, the more relevant the instruments are and the more precise the parameter estimates. Since we applied Bayesian estimation (see below), the *F* statistic is not available. However, it is possible to calculate the Wald statistic of regression estimates in Bayesian SEM for how strong an effect the instrument(s) has on independent variable(s)^90^, and a Wald statistic can be used to calculate the *F* statistic (see appendix in Maydeu-Olivares et al.^75^). Because the living distance between the grandparent and the grandchild was an ordinal variable with four categories, it was modelled using three dummy variables (i.e., in the same town, not in the same town but within 10 miles, further away in the UK, or overseas (= a reference category)) and defining it to have a direct effect on the outcome rather than a covariance with the outcome. This effectively means that instead of one instrument we have three instruments for grandparental investment. Note that AELEs were assumed to be exogenous variables, because the data used here provided no reasonable instruments for them. However, the interaction between endogenous grandparental investment and exogenous AELEs is likely also endogenous^91^. If grandparent’s living distance is a valid instrument for grandparental investment, then its interaction with AELEs works as a natural instrument for the endogenous interaction^91^ and one must use the same set of instruments for all endogenous predictors^92,93^. However, the relevance and exclusion criteria should be tested individually.

**Figure 1:**
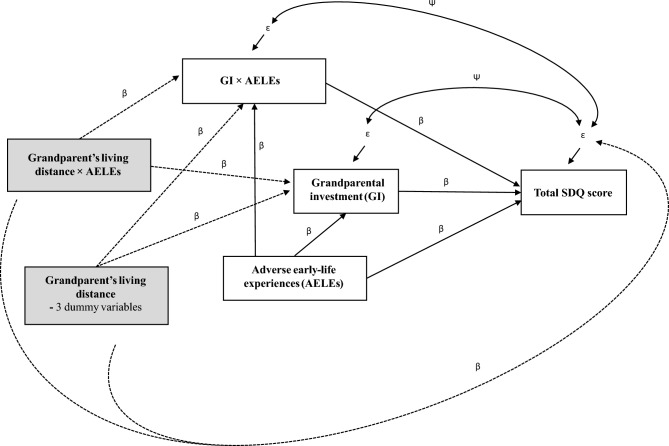
A graphical representation of instrumental variable regression model, fitted in structural equation modeling (SEM) framework. The main aim of the model is to examine whether grandparental investment (GI) moderated the influence of adverse early life experiences (AELEs) on the grandchild’s emotional and behavioral problems, measured by their total SDQ score. Boxes represent observed variables and single-headed straight arrows represent structural path coefficients (β’s) and the unobserved errors of observed variables (ε’s). Double-headed arrows represent the error covariances (Ψ) of the response variables. Instrumental variables are shown in grey boxes and they act as predictors of main independent variables in the analysis. A dotted single-headed arrows from the instruments to the grandparent’s investment and its interaction with AELEs shows the relevance criterion whereas the dotted single-headed arrow from the instruments to the outcome represents the exclusion criterion. This model was separately fitted for each grandparental type.

The instrumental variable regression model used here is illustrated in Fig. 1. The model was separately fitted for each grandparent type because combining all grandparent types into the same analysis would have included only those grandchildren who had all their grandparents alive. Because only those children who had at least one living grandparent were assigned to the original study (the authors of the current study could not influence this decision), selection bias arising from having no data on those grandchildren with no living grandparents might introduce some bias to the results^94^. In our case, this bias could not have been accounted for by using inverse probability weighting even if we had applied a frequentist approach because all conditional probabilities of grandparental investment are zero by default^95^. In addition to greatly reducing our sample size to 463 grandchildren, such a bias would probably have been more severe had we restricted to our sample to only those grandchildren having four living grandparents. Therefore, relevance and exclusion criteria were tested separately for each grandparent type using Wald tests^75^. The covariance between the errors of endogenous predictors and total SDQ score represents a test for endogeneity, caused by for example measurement error and shared omitted causes^72^. Instead of using a latent variable measuring grandparental investment as was done in Helle et al.^70^, we used an average of the four questions measuring grandparental investment. This was done because instrumental variable regression can handle measurement error in endogenous explanatory variables in addition to omitted variable bias^77^. Both grandparental investment and the grandchild’s AELEs were grand mean centered to make their interaction more interpretable.

To handle missing data in the variables at the item-scale (i.e., the questions used to measure grandparental investment and AELEs), we used multiple imputation and followed the guidelines given by von Hippel^96^ for the number of imputed data sets needed. By accepting a 5% change in the standard error of the estimates, we imputed 15 data sets using a variance covariance approach and unrestricted model with a Bayesian estimator^97^. The structural equation model was also fitted using Bayesian estimation and a Gibbs sampler for the Markov Chain Monte Carlo (MCMC) algorithm was used to draw posterior distribution for model parameters. The median of posterior distribution was used as a point estimate and the highest posterior density (HPD) was used for 95% (credibility) interval estimation. Non-informative normally distributed priors were used for structural regression coefficients [hyperparameters for prior mean and variances = N(0, 100^2^)] and non-informative inverse Wishart priors [IW(0, − 4)] for error variances and covariances. Two chains with a total of 20,000 iterations were run with a burn-in of 10,000 iterations. The convergence of MCMC chains was determined using a potential scale reduction factor that compared the estimated between-chains and within-chains variances for each parameter^98^. In general, values below 1.2 and 1.1 are considered to indicate good convergence of the chains. The maximum potential scale reduction factor was 1.003, suggesting appropriate convergence. Mplus version 8.7 was used for all data analyses^99^.

## Results

We first examined evidence for the assumptions of instrumental variable regression. The relevance criterion for a strong association between the instruments and their corresponding independent variables (*F* statistics > 10) was clearly satisfied for these data except for the interaction between grandparental investment and AELEs among PGFs, which fell just below this threshold (*F* statistics = 8.23) (see supplementary materials, Table S1). Moreover, the exclusion criterion of no association between the instruments and the outcome (i.e., total SDQ score) holds for all grandparent types (supplementary materials, Table S1). In addition, the covariance of the errors of independent variables and total SDQ score was non-zero, suggesting an endogeneity problem, but this was only among maternal grandmothers (MGMs: Wald χ^2^_2_ = 6.07, *P* = 0.048; MGFs: Wald χ^2^_2_ = 1.50, *P* = 0.47; PGMs: Wald χ^2^_2_ = 2.34, *P* = 0.31; PGFs: Wald χ^2^_2_ = 2.17, *P* = 0.34). Together, this supports causal interpretation of our results.

**Figure 2:**
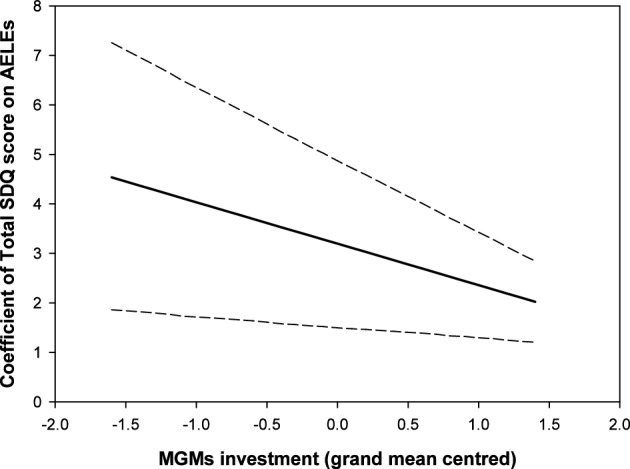
A graph showing how AELEs influence children’s total SDQ score depending on the level of their MGM’s investment. Short-dashed lines represent 95% credibility intervals of the posterior median.

The main results of the instrumental variable regression by grandparental type are shown in Table 1 (for the results of full instrumental variable models, please see supplementary materials, Table S2). Only the investment by MGMs moderated the harmful influence of multiple AELEs on a grandchild’s emotional and behavioral problems. That is, the estimate for the interaction between MGM’s investment and AELEs was negative and differed from zero, meaning that a one unit increase in grandparental investment decreased the positive effect of AELEs on a grandchild’s total SDQ score by, on average, 0.84 units (95% credibility intervals = − 1.48, − 0.21). In more detail, with the minimal investment from MGMs, every AELE increased a grandchild’s total SDQ score by, on average, 4.54 units (Fig. 2). In contrast, when MGM’s investment was at the highest level, a one-unit increase in AELE increased a grandchild’s total SDQ score by, on average, 2.02 units (Fig. 2). Note that no level of investment from MGMs observed in these data was able to fully erase the harmful influence of AELEs on children’s emotional and behavioral problems. For all other grandparent types, the interaction between grandparental investment and AELEs did not differ from zero (Table 1).

Because endogeneity was statistically detectable among MGMs only, the coefficients of instrumental variable regressions might have been inefficient (i.e., too large standard errors) for other grandparent types^92,93^. We therefore also ran regular regression models without instrumental variables, but the results remained qualitatively the same (see supplementary materials, Table S3).

## Discussion

Our findings are in line with the predictions derived from the grandmother hypothesis, sex-specific reproductive strategies and paternity uncertainty by providing the first causally more reliable evidence that investment by MGMs protected their grandchild from the negative influence of experiencing multiple AELEs in their early life. No such effect was found for other grandparent types. Although the investment by MGMs was able to cut the negative effect of AELEs on children’s emotional and behavioral problems by more than half, not even the highest level of investment from MGMs seen in these data was able to fully safeguard grandchildren from the negative effects of AELEs.

These findings strengthen our prior knowledge regarding the important role played by MGMs for their grandchildren^22,41^, even in unfavourable periods of their lives^62,70^. Although in contemporary affluent societies, the importance of grandfathers has been suggested to be increasing^100^, our results showed no support for a beneficial effect of grandfathers. These results corroborate recent findings indicating that MGMs did not reduce their investment in grandchildren like paternal grandparents did when the AELEs experienced by their grandchild increased^70^. MGMs, who can be certain of their relatedness to their grandchildren, may maximize their inclusive fitness by continuing to invest in their grandchildren despite the potential reduction in the reproductive value of the disadvantaged children. As shown in the current study, MGMs are, to some degree, able to buffer their grandchildren from suffering from increased emotional and behavioral problems when facing multiple AELEs. This, in turn, may improve the reproductive value of these children because, like in other social mammals, the adverse social environment experienced by the children during their development likely has consequences for their fitness through poorer long-term health and survival^101,102^ and higher probability of mental disorders in adulthood^103,104^. Furthermore, mental disorders reflected in the personality and behavior of an individual can be correlated with differential reproductive success^105–110^. More research is needed to examine whether the buffering effect of investment from MGMs on behavioral and emotional problems is strong enough to affect the long-term survival and reproductive outcomes of their grandchildren. In addition, although accounting for multiple adversities is advised as a way to effectively measure total environmental stress^64^, and earlier studies on this topic have used cumulative AELEs^65–67^, there could be distinct (i.e., non-additive) adverse early life events that affect specific developmental pathways and child outcomes that may show stronger or weaker, or adaptive or maladaptive, effects^6^.

The main strength of the current study is its reliance on instrumental variable regression^73,74^. This method is currently breaking its way into psychological and biological sciences^75–77^ and has potential to improve our ability to draw causal inferences from non-experimental data. This is an important asset because it would have been impossible for us to include all the potential confounders in the analysis, not least because many of them were unmeasured for the data set used here. For example, socioeconomic status of the grandparental and parental generations that are likely to lead to differences in resource availability to the families is one such confounder in studies on intergenerational relations^78^, and is unmeasured for the current data set. In addition, one could question the reliability of the measures of grandparental investment used here, either on conceptual grounds or because the grandchildren themselves provided the estimates of such investments. Although there are reasons to expect that children could actually be the most reliable source of information on variation of direct grandparental investment on them^43^, particularly when one is interested in comparing the investments between all four grandparent types, the instrumental variable approach can purge the effects of any measurement error. Finally, one does not need to worry about reverse causality (i.e., the possibility that emotional and behavioral problems of a child also affect grandparental investment, as well as vice versa) when applying valid instruments even if such effects existed.

Despite its potential for more reliable causal inference, instrumental variable regression is based on some assumptions (e.g., exclusion criterion) that cannot be fully empirically verified. While violations of those assumptions can severely bias causal inference drawn from such models^111^, one can still obtain important insights from instrumental variable regression by assuming instruments to be “plausibly exogenous”^112^. There is thus always a certain degree of uncertainty involved concerning causal claims and one has to also rely on subject knowledge to uphold those assumptions. For example, we cannot rule out the possibility that AELEs were truly exogenous, confounding its effects on grandparental investment and children’s emotional and behavioral problems. The potential, and in our case unsettled, selection bias due to the lack of grandchildren whose grandparents had all passed away in these data may have also influenced our results. There is also between-individual evidence that high grandparental investment may be associated with lower mortality^113,114^, although the findings are not unanimous^115^ and may vary by subpopulations^116^. Therefore, the selection bias owing to this latter process is unlikely to be strong.

In conclusion, the current study provides, to the best of our knowledge, the most causally informative and reliable research on whether grandparental investment moderates the harmful effects of cumulative AELEs on a child’s subsequent emotional and behavioral problems in contemporary wealthy society. The results are in accordance with expectations both from prior literature and from an evolutionary framework of cooperative breeding by showing that only MGMs, who generally invest the most in their grandchildren, were able to lessen the harmful effects AELEs have on their grandchild’s emotional and behavioral outcomes. Although it is recognised that the most relevant outcomes on wealthy western societies are psychological and behavioral wellbeing, further studies are needed to establish the long-term consequences of such investments on adulthood survival and reproductive output to understand the potential selective advantage of grandparental investments in this context. And because there are cultural differences in grandparental investment^117^, future studies should replicate our findings in other countries to validate their generalizability.

### Supplementary Information

Supplementary Tables.

## Data Availability

The data we used in this study are freely available from https://beta.ukdataservice.ac.uk/datacatalogue/studies/study?id=6075#!/details. Interested readers should be aware that, as the data are ‘safeguarded’ (https://www.ukdataservice.ac.uk/get-data/data-access-policy), a user will be required to register with the UK Data Service in order to access the data.
