# Supplementary material for: Investment by maternal grandmother buffers children against the impacts of adverse early life experiences
Source: Sci Rep. 2024 Mar 21;14:6815. doi: 10.1038/s41598-024-56760-5 (PMC10957867; doi:10.1038/s41598-024-56760-5)
Supplement: Supplementary file 1 — Supplementary Tables. [file 41598_2024_56760_MOESM1_ESM.pdf]

Supplementary information for:

Investment by maternal grandmother buffers children against the impacts of adverse early life experiences

Samuli Helle, Antti O. Tanskanen, David A. Coall, Gretchen Perry, Martin Daly & Mirkka Danielsbacka

Tests for the relevance and exclusion criteria

Table S1. Statistics for relevance and exclusion criteria by grandparental type. MGM, MGF, PGM, and PGF stand for maternal grandmothers, maternal grandfathers, paternal grandmothers and paternal grandfathers, respectively. Tests for relevance criterion are given separately for grandparental investment (GI) and its interaction with adverse early-life adversities (AELEs).

	Relevance criterion		Exclusion criterion	
	GI<br>*F* statistic	GI × AELEs<br>*F* statistic	χ^2_6	*p*-value
MGM	68.09	68.64	4.48	0.61
MGF	41.04	33.47	2.44	0.88
PGM	25.87	18.48	2.24	0.90
PGF	14.93	8.23	2.89	0.82

Full results for the instrumental variable models

Table S2. Full results of the instrumental variable models, estimated in Bayesian structural equation modeling framework. Note that grandparents were analysed in separate models. 95% C.I. denotes a 95% credibility interval of the posterior median of coefficients.

		95% C.I.	
	Median	Lower 2.5%	Upper 2.5%
Maternal grandmothers (n = 1,197)			
Total SDQ score regressed on			
Grandparental investment (GI)	2.476	0.923	4.012
Adverse early-life events (AELEs)	3.196	1.493	4.873
GI × AELEs	-0.839	-1.484	-0.212
Grandparental investment regressed on			
Adverse early-life events (AELEs)	-0.004	-0.083	0.075
Living distance (in the same town)	0.344	0.136	0.553
Living distance (not in the same town but within 10 miles)	0.729	0.529	0.922
Living distance (further away in the UK)	0.895	0.706	1.082
Living distance (in the same town) × AELEs	-0.061	-0.16	0.035
Living distance (not in the same town but within 10 miles) × AELEs	-0.02	-0.113	0.071
Living distance (further away in the UK) × AELEs	0.024	-0.061	0.111
GI × AELEs regressed on			
Adverse early-life events (AELEs)	1.969	1.773	2.158
Living distance (in the same town)	0.193	-0.244	0.63
Living distance (not in the same town but within 10 miles)	0.125	-0.309	0.562
Living distance (further away in the UK)	-0.085	-0.498	0.328
Living distance (in the same town) × AELEs	0.045	-0.187	0.277
Living distance (not in the same town but within 10 miles) × AELEs	0.587	0.357	0.825
Living distance (further away in the UK) × AELEs	1.013	0.797	1.229
Error covariances			
Total SDQ score with GI	-0.416	-0.79	-0.05
Total SDQ score with GI × AELEs	-0.35	-1.087	0.39
GI with GI × AELEs	0.517	0.468	0.569
Error variances			
Total SDQ score	31.316	28.643	34.237
Grandparental investment	0.315	0.290	0.342
GI × AELEs	1.464	1.335	1.603
Intercepts			
Total SDQ score	16.139	13.551	18.807
Grandparental investment	-0.631	-0.741	-0.521
GI × AELEs	3.115	2.881	3.351
Maternal grandfathers (n = 984)			
Total SDQ score regressed on			
Grandparental investment (GI)	1.037	-0.761	2.905

Adverse early-life events (AELEs)	1.547	-0.415	3.518
GI × AELEs	-0.227	-1.008	0.577
Grandparental investment regressed on			
Adverse early-life events (AELEs)	-0.102	-0.201	-0.001
Living distance (in the same town)	0.087	-0.167	0.347
Living distance (not in the same town but within 10 miles)	0.51	0.247	0.775
Living distance (further away in the UK)	0.728	0.481	0.98
Living distance (in the same town) × AELEs	0.054	-0.063	0.172
Living distance (not in the same town but within 10 miles) × AELEs	0.081	-0.033	0.2
Living distance (further away in the UK) × AELEs	0.08	-0.031	0.191
GI * AELEs regressed on			
Adverse early-life events (AELEs)	1.529	1.277	1.772
Living distance (in the same town)	-0.355	-0.93	0.227
Living distance (not in the same town but within 10 miles)	-0.445	-1.007	0.125
Living distance (further away in the UK)	-0.349	-0.898	0.186
Living distance (in the same town) × AELEs	0.444	0.155	0.752
Living distance (not in the same town but within 10 miles) × AELEs	0.999	0.718	1.291
Living distance (further away in the UK) × AELEs	1.147	0.879	1.44
Error covariances			
Total SDQ score with GI	-0.295	-0.777	0.179
Total SDQ score with GI × AELEs	-0.483	-1.577	0.569
GI with GI × AELEs	0.632	0.565	0.701
Error variances			
Total SDQ score	31.316	28.412	34.440
Grandparental investment	0.378	0.344	0.413
GI × AELEs	1.922	1.744	2.104
Intercepts			
Total SDQ score	13.788	10.663	16.888
Grandparental investment	-0.551	-0.698	-0.406
GI × AELEs	2.908	2.582	3.233
Paternal grandmothers (*n* = 1,047)			
Total SDQ score regressed on			
Grandparental investment (GI)	1.699	-0.7	4.229
Adverse early-life events (AELEs)	2.209	-0.164	4.739
GI × AELEs	-0.599	-1.711	0.447
Grandparental investment regressed on			
Adverse early-life events (AELEs)	-0.057	-0.154	0.05
Living distance (in the same town)	0.151	-0.081	0.391
Living distance (not in the same town but within 10 miles)	0.584	0.343	0.827

Living distance (further away in the UK)	0.572	0.345	0.803
Living distance (in the same town) × AELEs	0.008	-0.113	0.117
Living distance (not in the same town but within 10 miles) × AELEs	-0.022	-0.155	0.093
Living distance (further away in the UK) × AELEs	0.043	-0.071	0.149
GI × AELEs regressed on			
Adverse early-life events (AELEs)	1.719	1.405	2.024
Living distance (in the same town)	-0.099	-0.702	0.491
Living distance (not in the same town but within 10 miles)	0.106	-0.508	0.709
Living distance (further away in the UK)	-0.099	-0.687	0.471
Living distance (in the same town) × AELEs	0.221	-0.109	0.577
Living distance (not in the same town but within 10 miles) × AELEs	0.463	0.064	0.806
Living distance (further away in the UK) × AELEs	0.753	0.431	1.078
Error covariances			
Total SDQ score with GI	-0.297	-0.884	0.278
Total SDQ score with GI × AELEs	-0.006	-1.498	1.495
GI with GI × AELEs	0.675	0.61	0.748
Error variances			
Total SDQ score	31.636	28.716	35.425
Grandparental investment	0.361	0.331	0.394
GI × AELEs	2.116	1.920	2.333
Intercepts			
Total SDQ score	14.793	10.909	18.914
Grandparental investment	-0.419	-0.548	-0.289
GI × AELEs	3.004	2.688	3.328
Paternal grandfathers (*n* = 817)			
Total SDQ score regressed on			
Grandparental investment (GI)	1.73	-1.349	4.853
Adverse early-life events (AELEs)	1.921	-1.263	5.129
GI × AELEs	-0.45	-1.987	1.019
Grandparental investment regressed on			
Adverse early-life events (AELEs)	-0.024	-0.123	0.078
Living distance (in the same town)	0.282	0.004	0.556
Living distance (not in the same town but within 10 miles)	0.709	0.427	0.992
Living distance (further away in the UK)	0.625	0.351	0.9
Living distance (in the same town) × AELEs	-0.051	-0.171	0.067
Living distance (not in the same town but within 10 miles) × AELEs	-0.084	-0.207	0.036
Living distance (further away in the UK) × AELEs	-0.002	-0.117	0.118
GI × AELEs regressed on			

Adverse early-life events (AELEs)	1.771	1.526	2.026
Living distance (in the same town)	0.176	-0.474	0.841
Living distance (not in the same town but within 10 miles)	0.461	-0.204	1.139
Living distance (further away in the UK)	0.152	-0.491	0.803
Living distance (in the same town) × AELEs	0.035	-0.269	0.337
Living distance (not in the same town but within 10 miles) × AELEs	0.198	-0.107	0.501
Living distance (further away in the UK) × AELEs	0.523	0.205	0.824
Error covariances			
Total SDQ score with GI	-0.513	-1.316	0.243
Total SDQ score with GI × AELEs	-0.551	-2.754	1.638
GI with GI × AELEs	0.725	0.643	0.815
Error variances			
Total SDQ score	30.812	27.012	35.549
Grandparental investment	0.399	0.361	0.439
GI × AELEs	2.284	2.042	2.535
Intercepts			
Total SDQ score	14.132	9.071	19.414
Grandparental investment	-0.425	-0.580	-0.269
GI × AELEs	2.839	2.468	3.209

Selected results of regular regression models

Table S3. Selected results of regular regression models on the influence of grandparental investment on grandchild’s total SDQ score, moderated by adverse early life experiences for different grandparental types. 95% C.I. denotes a 95% credibility interval of the posterior median of coefficients. For a positive posterior median, one-tailed probability gives the proportion of posterior distribution that is below zero, and for a negative posterior median the proportion of posterior distribution that is above zero is given.

	median	95% C.I.	One-tailed probability
MGMs			
Granparent's investment (GI)	0.826	0.063, 1.592	0.017
AELEs	2.147	1.222, 3.092	0.000
GI × AELEs	-0.440	-0.789, -0.095	0.007

MGFs			
Granparent's investment (GI)	0.408	-0.369, 1.175	0.150
AELEs	1.478	0.613, 2.353	0.000
GI × AELEs	-0.208	-0.558, 0.131	0.118
PGMs			
Granparent's investment (GI)	-0.054	-0.905, 0.834	0.452
AELEs	0.918	0.070, 1.771	0.017
GI × AELEs	-0.040	-0.396, 0.324	0.413
PGFs			
Granparent's investment (GI)	-0.033	-0.921, 0.874	0.472
AELEs	1.101	0.248, 1.959	0.006
GI × AELEs	-0.100	-0.480, 0.288	0.303

Descriptive statistics for the variables used in the study

Table S4. Descriptive statistics (%) for the variables used to measure grandparental investment by grandparental type. MGM, MGF, PGM, and PGF stand for maternal grandmothers, maternal grandfathers, paternal grandmothers and paternal grandfathers, respectively.

	MGM	MGF	PGM	PGF
"Do they give you money or help in any other way?" (%)				
Never	9.19	14.02	14.04	17.63
Occasionally	34.42	31.81	35.53	35.01
Usually	54.30	49.29	46.04	42.47
Missing	2.09	4.88	4.39	4.90
"How often do you see them?" (%)				
Never	5.85	7.93	8.02	11.63
Several times a year	36.76	36.89	45.75	44.68
Twice a week	36.09	33.13	32.00	30.72
Daily	19.63	15.55	8.69	6.49
Missing	1.67	6.50	5.54	6.49
"How often do your grandparents look after you?" (%)				
Never	29.07	34.96	40.97	46.27
Several times a year	39.35	34.76	36.10	32.56
Once a week or so	24.90	22.15	17.48	16.28
Every day	4.76	3.86	1.34	0.98
Missing	1.92	4.27	4.11	3.92
"How much can you depend on your grandparent to be there when you really need him/her?" (%)				
Not at all	10.61	14.13	16.52	20.20

A little	14.04	15.65	16.91	16.77
Sometimes	22.06	21.34	24.16	23.99
A lot	51.55	44.51	38.01	34.64
Missing	1.75	4.37	4.39	4.41

Mplus code for performing instrumental variable regression using structural equation modeling (SEM)

We first performed multiple imputation to handle missing data in variables. Note that imputation was performed at the item-level (i.e., before actual analysis variables were constructed).

```
DATA:
     FILE IS C:\original_data.txt;
     TYPE IS IND;
  VARIABLE:
     NAMES ARE
       q15_1 q15_2 q15_3 q15_4
       q26_1 q26_2 q26_3 q26_4
       q27_1 q27_2 q27_3 q27_4
       q38_1 q38_2 q38_3 q38_4
       f2_1 f2_6 f2_7 f2_8 f2_16 f2_22
       Tot_SDQ d div
       gp1 gp2 gp3 gp4
       ;
     USEVARIABLES ARE
       q15_1 q15_2 q15_3 q15_4
       q26_1 q26_2 q26_3 q26_4
       q27_1 q27_2 q27_3 q27_4
       q38_1 q38_2 q38_3 q38_4
       f2_1 f2_6 f2_7 f2_8 f2_16 f2_22
       Tot_SDQ d div
       ;
     AUXILIARY = gp1 gp2 gp3 gp4;
     MISSING ARE ALL(-99);
  DATA IMPUTATION:
     IMPUTE = q15_1 (c) q15_2 (c) q15_3 (c) q15_4 (c)
              q16_1 (c) q16_2 (c) q16_3 (c) q16_4 (c)
              q26_1 (c) q26_2 (c) q26_3 (c) q26_4 (c)
              q27_1 (c) q27_2 (c) q27_3 (c) q27_4 (c)
              q38_1 (c) q38_2 (c) q38_3 (c) q38_4 (c)
              f2_1 (c) f2_6 (c) f2_7 (c) f2_8 (c) f2_16 (c) f2_22 (c) d (c)
              Tot_SDQ div (c);
     NDATASETS = 15;
     VALUES = q15_1 (1-4) q15_2 (1-4) q15_3 (1-4) q15_4 (1-4)
              q16_1 (0-3) q16_2 (0-3) q16_3 (0-3) q16_4 (0-3)
              q26_1 (1-4) q26_2 (1-4) q26_3 (1-4) q26_4 (1-4)
              q27_1 (1-4) q27_2 (1-4) q27_3 (1-4) q27_4 (1-4)
              q38_1 (1-3) q38_2 (1-3) q38_3 (1-3) q38_4 (1-3)
              f2_1 (0-1) f2_6 (0-1) f2_7 (0-1) f2_8 (0-1)
```

```
                    f2_16 (0-1) f2_22 (0-1) Tot_SDQ (0-33) d (0-1) div (0-1);
    SAVE = imdata*.dat;
  ANALYSIS:
    TYPE = BASIC;
     PROCESSORS = 2;
     BSEED=267;
  OUTPUT:
    TECH8 TECH9;
```

The code below performs instrumental variable regression using SEM and Bayesian estimation on the imputed data sets. Please note that the code was ran separately for each grandparent type.

```
  FILE IS imdatalist.dat;
  TYPE = IMPUTATION;
VARIABLE:
  NAMES ARE
  Q15_1
  Q15_2
  Q15_3
  Q15_4
  Q16_1
  Q16_2
  Q16_3
  Q16_4
  Q26_1
  Q26_2
  Q26_3
  Q26_4
  Q27_1
  Q27_2
  Q27_3
  Q27_4
  Q38_1
  Q38_2
  Q38_3
  Q38_4
  F2_1
  F2_6
  F2_7
  F2_8
  F2_16
  F2_22
  TOT_SDQ
  SEX
  D
  DIV
  GP1
```

```
   GP2
   GP3
   GP4
   ;
 USEVARIABLES ARE
    Tot_SDQ
   Invest
   aele
   aeleinv
   dis1-dis3
   z1-z3
   ;
  USEOBSERVATIONS = GP1 EQ 1;
    MISSING ARE *;
DEFINE:
   Invest = MEAN(q38_1 q15_1 q26_1 q27_1);
   aele=F2_1+F2_6+F2_7+F2_8+F2_16+F2_22+d+div;
   aeleinv=Invest*aele;

  dis1 = 0;
  IF q16_1 eq 1 then dis1 = 1;
  dis2 = 0;
  IF q16_1 eq 2 then dis2 = 1;
  dis3 = 0;
  IF q16_1 eq 3 then dis3 = 1;

   z1=risk*dis1;
   z2=risk*dis2;
   z3=risk*dis3;

 CENTER Inv_mm risk (grandmean);

ANALYSIS:
  ESTIMATOR = BAYES;
  PROCESSOR=2;
  BSEED=2381;
  FBITER=20000;

MODEL:

    Invest ON aele;

    Tot_SDQ ON Invest (p1)
            aele (p2)
            aeleinv (p3);

    Invest ON dis1 (d1)
                 dis2 (d2)
                 dis3 (d3);
```

```
    aeleinv ON z1 (d4)
                    z2 (d5)
                    z3 (d6);

   Tot_SDQ WITH Invest (c1);
   Tot_SDQ WITH aeleinv (c2);
   aeleinv WITH Invest;

!    Tot_SDQ ON dis1 (e1);                    # Define paths to test relevance criterion
!    Tot_SDQ ON dis2 (e2);                    # Remove “!” to run the code
!    Tot_SDQ ON dis3 (e3);

!    Tot_SDQ ON z1 (e4);
!    Tot_SDQ ON z2 (e5);
!    Tot_SDQ ON z3 (e6);

  MODEL TEST:

!      0=d1;                        # Test of the relevance criterion
!      0=d2;
!      0=d3;
!      0=d4;
!      0=d5;
!      0=d6;

!      0=c1;                        # Test of endogeneity
!      0=c2;

!       0=e1;                       # Test of exclusion criterion
!       0=e2;
!       0=e3;
!       0=e4;
!       0=e5;
!       0=e6;

MODEL CONSTRAINT:                     # Estimates used in Figure 2

   NEW (est1-est31);
   est1=p2+p3*(-1.6);
   est2=p2+p3*(-1.5);
   est3=p2+p3*(-1.4);
   est4=p2+p3*(-1.3);
   est5=p2+p3*(-1.2);
   est6=p2+p3*(-1.1);
   est7=p2+p3*(-1.0);
   est8=p2+p3*(-0.9);
   est9=p2+p3*(-0.8);
   est10=p2+p3*(-0.7);
   est11=p2+p3*(-0.6);
   est12=p2+p3*(-0.5);
```

```
    est13=p2+p3*(-0.4);
    est14=p2+p3*(-0.3);
    est15=p2+p3*(-0.2);
    est16=p2+p3*(-0.1);
    est17=p2+p3*0;
    est18=p2+p3*0.1;
    est19=p2+p3*0.2;
    est20=p2+p3*0.3;
    est21=p2+p3*0.4;
    est22=p2+p3*0.5;
    est23=p2+p3*0.6;
    est24=p2+p3*0.7;
    est25=p2+p3*0.8;
    est26=p2+p3*0.9;
    est27=p2+p3*1.0;
    est28=p2+p3*1.1;
    est29=p2+p3*1.2;
    est30=p2+p3*1.3;
    est31=p2+p3*1.4;

OUTPUT:
     SAMPSTAT;
     CINT(HPD);
```
